# Clinical evaluation of a combination therapy of imepitoin with phenobarbital in dogs with refractory idiopathic epilepsy

**DOI:** 10.1186/s12917-017-0957-z

**Published:** 2017-01-25

**Authors:** Jasmin Neßler, Chris Rundfeldt, Wolfgang Löscher, Draginja Kostic, Thomas Keefe, Andrea Tipold

**Affiliations:** 1 Department of Small Animal Medicine and Surgery, University of Veterinary Medicine, 30559 Hannover, Germany; 2 Department of Pharmacology, Toxicology and Pharmacy, University of Veterinary Medicine Hannover, 30559 Hannover, Germany; 3 Drug-Consulting Network, 01445 Coswig, Germany; 4 Center for Systems Neuroscience, 30559 Hannover, Germany; 5 Department of Environmental & Radiological Health Sciences, College of Veterinary Medicine & Biomedical Sciences, Colorado State University, Fort Collins, USA

**Keywords:** Idiopathic epilepsy, Combination treatment, Potassium bromide, Levetiracetam, Anticonvulsant, Tonic-clonic seizures

## Abstract

**Background:**

Imepitoin was tested as a combination treatment with phenobarbital in an open-label mono-centre cohort study in dogs with drug-resistant epilepsy. Diagnosis of idiopathic epilepsy was based on clinical findings, magnetic resonance imaging and cerebrospinal fluid analysis. Three cohorts were treated. In cohort A, dogs not responding to phenobarbital with or without established add-on treatment of potassium bromide or levetiracetam were treated add-on with imepitoin, starting at 10 mg/kg BID, with titration allowed to 30 mg/kg BID. In cohort B, the only difference to cohort A was that the starting dose of imepitoin was reduced to 5 mg/kg BID. In cohort C, animals not responding to imepitoin at >20 mg/kg BID were treated with phenobarbital add-on starting at 0.5 mg/kg BID.

**Results:**

The add-on treatment resulted in a reduction in monthly seizure frequency (MSF) in all three cohorts. A reduction of ≥50% was obtained in 36-42% of all animals, without significant difference between cohorts. The lower starting dose of 5 mg/kg BID imepitoin was better tolerated, and an up-titration to on average of 15 mg/kg BID was sufficient in cohort A and B. In cohort C, a mean add-on dose of 1.5 mg/kg BID phenobarbital was sufficient to achieve a clinically meaningful effect. Six dogs developed a clinically meaningful increase in MSF of ≥ 50%, mostly in cohort A. Neither imepitoin nor phenobarbital add-on treatment was capable of suppressing cluster seizure activity, making cluster seizure activity an important predictor for drug-resistance.

**Conclusion:**

A combination treatment of imepitoin and phenobarbital is a useful treatment option for a subpopulation of dogs with drug-resistant epilepsy, a low starting dose with 5 mg/kg BID is recommended.

## Background

Idiopathic epilepsy is defined as chronic spontaneous seizure activity and can be sub-classified into three sub-groups: genetic epilepsy, suspected genetic epilepsy, and epilepsy of unknown cause, in which the nature of the underlying cause is unknown and no indication of structural epilepsy occurs [1]. Idiopathic epilepsy is diagnosed, if the dog experienced the first seizure activity at an age of 6 months up to 6 years, if reasons for structural epilepsy or reactive seizures (due to extracranial causes) are excluded and if the dog does not show neurological abnormalities in the interictal period. Magnetic resonance imaging (MRI) and cerebrospinal fluid (CSF) analysis showing a lack of structural pathology supports the diagnosis [1, 2]. Familial predisposition or predisposition in some dog breeds is described [3]. In these breeds, such as Australian Shepherds and Border Collies, a severe course of the disease is seen [1, 4, 5]. The treatment of epilepsy is hampered by the fact that 20-40% of dogs with idiopathic epilepsy may be treatment-resistant to one or several antiepileptic medications [3]. In these cases, an additional medication is recommended (add-on treatment).

Imepitoin is a partial agonist at the benzodiazepine recognition site of the γ-amino butyric acid (GABA_A_) receptor that was developed as an antiepileptic (antiseizure) drug (AED) for canine idiopathic epilepsy [6–9]. Since its introduction in 2013, imepitoin has become one of the two AEDs with the highest level of evidence for efficacy for the treatment of generalized tonic-clonic seizures, with an improved safety profile compared to phenobarbital [10, 11]. Imepitoin currently has no specific add-on therapy label for combination treatment with other AEDs. So far, only one pilot study [12] evaluated the safety and efficacy of imepitoin administered add-on to phenobarbital monotherapy. No severe adverse drug reactions of the combination and a favorable outcome comparable to add-on treatment with potassium bromide were reported.

To obtain controlled information on the safety and efficacy of combination treatment of imepitoin with phenobarbital with or without other antiepileptic co-medications in patients with drug resistance to previous treatment, a single center, prospective, controlled, open label, cohort study was initiated.

## Methods

The study was conducted in 2013–2015 as a single center, prospective, controlled, open label, cohort study in client-owned dogs with drug resistant idiopathic epilepsy. The study was conducted according to the ethical rules of the University of Veterinary Medicine, Hannover, Germany, with approval by the institutional animal welfare officer, and with written informed consent of the owners.

### Study design

The study was designed to reflect the population of dogs with drug-resistant epilepsy, as presenting in a veterinary neurology referral center. In a first cohort (cohort A), client owned dogs suffering from insufficient seizure control from treatment with phenobarbital, with or without co-medication with potassium bromide and/or levetiracetam, were included. These patients were treated add-on with imepitoin, using the labelled starting dose of 10 mg/kg imepitoin, administered BID, with titration steps allowed to 30 mg/kg BID. After collection of cohort A, two further cohorts were added. In cohort B, the safety and efficacy of a lower starting add-on dose of imepitoin of 5 mg/kg BID was evaluated, corresponding to the protocol used previously evaluating the safety and efficacy of imepitoin add-on to phenobarbital [12]. All other in- and exclusion criteria were identical to cohort A. This change was made due to observation of increased seizure frequency or cluster seizures in four animals upon add-on administration of imepitoin in cohort A. In addition, transient ataxia was observed in some of the patients in cohort A following initiation of add-on treatment with imepitoin. Cohort C was designed to include patients not responding to imepitoin, with or without add-on treatment with levetiracetam, to be treated add-on with phenobarbital. For this cohort, a low starting dose of 0.5 mg/kg BID phenobarbital was selected to avoid described adverse drug reactions of phenobarbital [3].

The diagnosis of idiopathic epilepsy was based on a clinical and neurological examination as described by Vandevelde et al. [13], and age of onset of epilepsy between 6 months and 6 years [2]. The diagnosis was verified using blood work, MRI, and CSF analysis (Tier II confidence level for diagnosis of idiopathic epilepsy) [2]. For inclusion in cohort A or B, animals had to be treated with phenobarbital for ≥ 8 weeks, or in case of potassium bromide add-on treatment, for ≥ 3 months prior inclusion, with proven therapeutic serum levels for phenobarbital (10–35 μg/ml) and potassium bromide (0.5-2.5 mg/ml). Levetiracetam was allowed, either as baseline medication, or as pulse therapy at times of increased seizure activity. For inclusion in cohort C, at least 6 weeks treatment with imepitoin at the upper therapeutic dose range (>20 mg/kg BID) was required. For all animals, at least 6 weeks seizure diary recordings were required, demonstrating insufficient seizure control as defined by at least 1 generalized tonic-clonic seizure within 6 weeks prior to inclusion despite serum levels of applied AEDs within known therapeutic range [14], or treatment with imepitoin at a dose of >20 mg/kg BID (=baseline). Dogs with cluster seizures as defined by more than one seizure within less than 24 h [1] or status epilepticus as defined by >5 min continuous epileptic seizure activity, or occurrence of two or more epileptic seizures with incomplete recovery of consciousness between seizures [1] were allowed to be included. Optional seizure diaries exceeding the 6 weeks baseline period were recorded as seizure history.

Dogs were specifically excluded for enrolment if their case history included any of the following: Signs of severe hepatic, renal or cardiac insufficiency, structural epilepsy or reactive seizures, severe, unacceptable side effects due to current/ongoing treatment, pregnancy or lactation, administration of further AEDs, other than phenobarbital, potassium bromide, levetiracetam or imepitoin, or body weight below 5 kg.

After cohort assignment, add-on treatment was started with 10 mg/kg or 5 mg/kg BID imepitoin (cohort A and B) or 0.5 mg/kg BID phenobarbital (cohort C). Owners were advised to generate a seizure diary, and to observe the animals for potential adverse drug reactions. Following inclusion, follow-up visits were scheduled after 4 weeks, 12 weeks, and 24 weeks. During each visit, the seizure diary was reviewed and seizures as well as reported suspected adverse drug reactions were transferred to the study files. A general and neurological examination was conducted, and blood samples were taken for complete blood cell count, biochemistry profile, and for phenobarbital and potassium bromide serum levels.

**Fig. 1:**
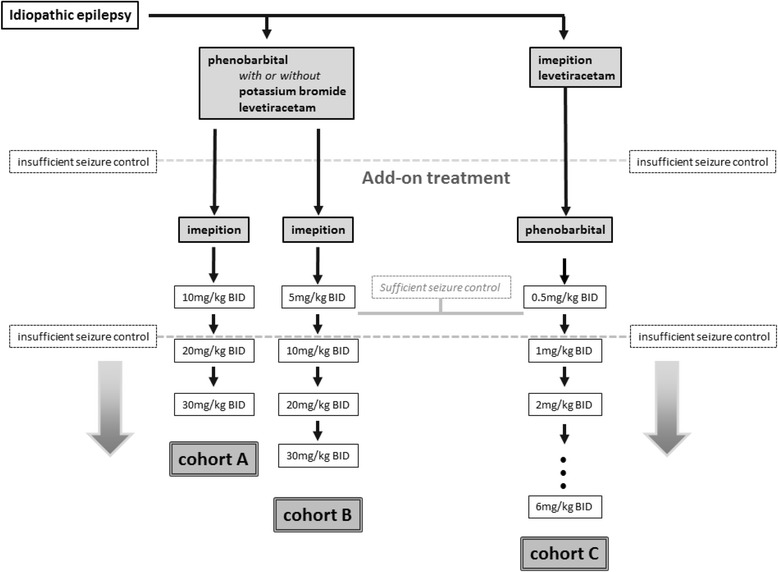
Study flow-chart

If seizure control was insufficient as observed upon occurrence of seizure or cluster activity, the dose of the add-on medication was increased, while the initial treatment stayed unchanged. Scheduled visits, unscheduled visits and phone contacts were utilized for dose adjustments. Any dose adjustment was done as case-by-case decision, taking into account the baseline seizure frequency and seizure events occurring after initiation of add-on treatment. In cohort A and B, dose adjustments to up to 30 mg/kg BID in two to three steps were usually done after at least one week of add-on treatment at the previous dose level. In cohort C, the phenobarbital dose was adjusted after at least two weeks of treatment at the previous dose level, to allow steady state to be reached. A top dose of 6 mg/kg BID was allowed, but not utilized (Fig. 1). After the last study visit, owners were allowed to continue add-on treatment, but were not followed further. Premature termination was permitted, if seizure reduction was still insufficient despite the dogs receiving the maximum dose of the add-on treatment (imepitoin 30 mg/kg BID, phenobarbital 6 mg/kg BID), if phenobarbital serum levels were above 35 μg/ml, if severe side effects occurred or if the owner elected euthanasia because of perceived lack of quality of life. Owners were allowed to withdraw consent and to leave the study at any time.

### Statistical evaluation of efficacy and safety

Statistical evaluation was performed by independent persons (CR, TK) to avoid a potential bias effect by clinical investigators. All animals of each cohort which had received at least one dose of add-on medication were included in the primary evaluation of efficacy and safety. Cohorts A and B as well as all three cohorts were also pooled. In addition, the animals completing the full add-on treatment period were evaluated separately.

The primary efficacy variable was the reduction in monthly seizure frequency (MSF) during treatment compared to the defined baseline period for the individual dog. To calculate MSF, the seizures recorded during the baseline period were counted. All documented generalized tonic-clonic seizures, seizures during cluster, or status events which had occurred during the 42 days of baseline period were counted. Monthly seizure frequencies were then calculated on the basis of one month notionally equaling 28 days. For the add-on treatment period, the same procedure was utilized, counting all seizure events from first day of add-on treatment to study termination. In addition, the monthly frequency of days with cluster seizures (MCD) was calculated, counting all days with more than one seizure event occurring on that day as cluster day. This definition was selected instead of the definition of a cluster event as more than one seizure occurring within less than 24 h, since for some seizure events adequate time information was not available.

Because of the skewness of the distribution of seizure frequency related measures, geometric means (GM) and 95% confidence interval (CI) were calculated for MSF and MCD for baseline and add-on treatment period. Because MCD had some zero values, the smallest non-zero value of MCD was added to each MCD value before log-transformation. This offset was subtracted again from the antilog of the mean and 95% and CI, to obtain the correct GM and 95% CI. The percent decrease in MSF was calculated for each dog individually as 100 x (1 – R) where R is the ratio of the MSF during treatment to the MSF during baseline. To obtain the estimated percent decrease + confidence interval for each cohort and combined cohorts, in a first step the GM and its 95% CI was calculated for R. The estimated percent decrease in MSF between the baseline and treatment periods and its CI were then obtained by applying the expression 100x (1 – R) to the GM of R and to the upper and lower CI endpoints, respectively, of the CI for R. To calculate the percent decrease in MCD, the same procedure was utilized, with the exception that the smallest observed MCD interval had to be added to all baseline and treatment results, to enable inclusion of animals which had had no cluster days during the baseline period.

For MSF and MCD, the paired *t*-test was applied to the corresponding log-transformed data values separately for each cohort and the combinations of cohorts (i.e., cohort A + B and all 3 cohorts combined). *P* < 0.05 was considered significant, while *p* < 0.1 was accepted as statistical trend. As confirmatory analysis, the data on MSF for cohorts A to C were statistically evaluated via repeated-measures analysis of variance of log-transformed values with main effects for cohort and period and the interaction between cohort and period, followed by Fisher’s least-significant-different comparisons per cohort of the mean difference between the baseline and treatment periods. Frequencies of events were compared using Chi-square test. Continuous baseline variables were compared using analysis of variance with Bonferoni’s multiple comparison test.

The safety of the study treatments was assessed on the basis of suspected adverse drug reactions (ADRs) reported for all cases enrolled in the study (i.e., during both the titration and evaluation of efficacy phases) following classification according to the VeDDRA list of preferred terms of the system organ classes (European Medicines Agency 2004; 2011). Laboratory data were evaluated based on standard descriptive statistics and were reviewed for conspicuous individual changes.

## Results

In total 34 dogs were enrolled, with 16 dogs in cohort A, 11 dogs in cohort B, and 7 dogs in cohort C. All animals met all of the inclusion criteria at screening, and none of the dogs met an exclusion criterion. There was a predominance of male animals in all cohorts, with in total 26 males and 8 females. While less than 50% of males were neutered, 7 out of 8 females were neutered. The body weight of included animals was in the range of 7.3 kg - 58.4 kg, and there was no significant difference in body weight distribution between cohorts. Nineteen different breeds were enrolled, with Australian Shepherd, Border Collie and Belgium Shepherd representing in total 14 of 34 dogs. Because the study was a cohort study, the animals were not randomized with regard to their baseline characteristics. Instead, animals were sequentially included upon their appearance in the clinics. As a result, animals differed somewhat in their demographics and baseline characteristics (Tables 1, 2).

**Table 1:** Demography

Parameter		Cohort A *N* = 16	Cohort B *N* = 11	Cohort C *N* = 7	Total *N* = 34	Group comparison
Sex (n)	male/neut. female/neut.	11/3 5/5	9/4 2/2	6/4 1/0	26/11 8/7	*p =* 0.595^1^
Age [years] at onset	Mean ± SEM Range	2.92 ± 0.33 0.75-5.62	3.99 ± 0.57 1.33-6.77	1.79 ± 0.41 0.83-3.50	2.93 ± 0.28 0.75-6.76	*p* = 0.018^2^ B > C*
Age [years] at inclusion	Mean ± SEM Range	5.52 ± 0.63 1.02-11.18	6.06 ± 0.70 1.81-8.89	3.19 ± 0.88 0.97-6.99	5.21 ± 0.44 0.97-11.81	*p =* 0.052^2^
Weight [Kg]	Mean ± SEM Range	26.11 ± 3.04 7.30-26.11	22.15 ± 2.17 7.40-30	23.80 ± 6.10 10.50-58.4	24.36 ± 1.98 7.3-58.4	*p =* 0.626^2^

^1^Chi-Square test comparing cohorts A, B and C

^2^Analysis of variance, with Bonferroni's multiple comparison test. *B > C, mean difference 2.2 years, confidence interval 0.34 to 4.06 years

**Table 2:** Baseline characteristics

Parameter	Dimension	Cohort A	Cohort B	Cohort C	Group comparison
Seizure activity related parameter					
Duration of epilepsy at inclusion in days	Geometric Mean (95% CI)	642 (372–1110)	556.6 (313–991)	292.1 (93–915)	*p =* 0.334^1^
	Mean ± SEM	950 ± 183	755 ± 180	512 ± 193	
Monthly seizure frequency (MSF)	Geometric Mean (95% CI)	4.06 (2.93-5.61)	3.73 (1.79-7.77)	3.54 (2.45-5.10)	*p =* 0.602^1^
Monthly cluster day (MCD)	Geometric Mean (95% CI)	0.62 (0.26-1.31)	0.48 (0.10-1.37)	0.51 (0.10-1.53)	*p =* 0.732^1^
Animals with cluster in baseline or history	m/n (%)	14/16 (87.5%)	6/11 (54.5%)	6/7 (85.7%)	*p =* 0.113^3^
Animals with status in baseline or history	m/n (%)	4/16 (25%)	1/11 (9.1%)	2/7 (28.6%)	*p =* 0.509^3^
Animals without cluster or status in baseline or history	m/n (%)	2/16 (12.5%)	5/11 (45.5%)	1/7 (14.3%)	*p =* 0.113^3^
Medication related parameter					
Dose of phenobarbital (A, B) or imepitoin (C) at inclusion in mg/kg per day	Mean ± SEM Range	9.49 ± 0.59 5.0-13.2	8.16 ± 0.59 4.0-11.4	55.0 ± 4.30 40.0-76.0	*p =* 0.138^2^
Frequency of dogs with KBr at inclusion	m/n (%)	7/16 (43.8%)	2/11 (18.2%)	0/7 (0%)	*p =* 0.069^3^ *p =* 0.166^4^
Frequency of dogs with any 2^nd^permanent antiepileptic at inclusion	m/n (%)	9/16 (56.3%)	3/11 (27.3%)	0/7 (0%)	*p =* 0.027^3^ *p =* 0.137^4^
Frequency of dogs with levetiracetam pulse therapy in baseline	m/n (%)	1/16 (6.3%)	4/11 (36.4%)	2/7 (28.6%)	*p =* 0.138^3^ *p =* 0.048^4^
Add-on dose of imepitoin (A, B) or phenobarbital (C) at inclusion (mg/kg per day)	Mean ± SEM Range	23.5 ± 1.44 17.6-40.0	10.8 ± 0.47 8.6-14.8	1.2 ± 0.06 0.9-1.4	

^1^Analysis of variance, with Bonferroni's multiple comparison test

^2^t-Test comparing cohort A and B ^3^Chi-Square test comparing cohorts A, B and C ^4^Chi-Square test comparing cohort A and B

There was a trend that animals from cohort B appeared to suffer from less severe disease as compared to cohort A. Disease related differences were: the duration of epilepsy prior to inclusion was shorter and the fraction of animals which had suffered from status or cluster seizures was lower in cohort B compared to cohort A. In fact, in cohort A nearly all animals (87.5%) had suffered status or cluster activity prior to inclusion, while in cohort B only 54.5% of dogs had suffered the same (Table 2). Cohort A and B also differed in the baseline medication (Table 2). The administered dose of phenobarbital was slightly lower in cohort B compared to A, and the proportion of dogs which were also treated with a 2^nd^ baseline medication, was 56.3% in cohort A and only 27.3% in cohort B.

Cohort C was again different, with much shorter duration of disease prior to inclusion and with no 2^nd^ permanent baseline antiepileptic at inclusion. However, in cohort C the rate of patients with cluster seizure activity was comparable to cohort A, with 85.7% of animals having experienced cluster seizures prior to inclusion.

### Antiepileptic activity

**Fig. 2:**
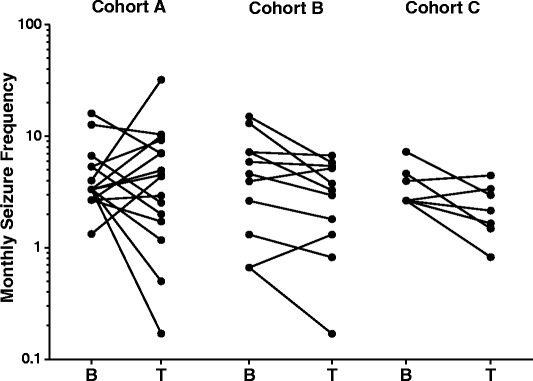
Development of monthly seizure frequency during baseline (B) and add-on treatment (T). Individual animal values are displayed

**Fig. 3:**
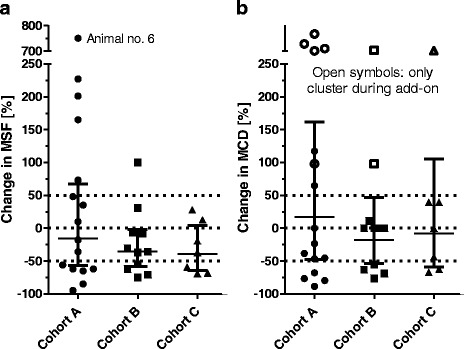
Change in monthly seizure frequency (MSF, **a**) and in monthly cluster days (MCD, **b**) for each individual animal [%] and estimated % reduction in MSF/MCD with 95% CI. Animals below the lower dotted line (>50% reduction) are responders, animals above the upper dotted line had >50% increase in MSF or MCD. Dogs between the dotted lines (−50% to +50%) had no clinically meaningful change of MSF or MCD. Note, that 8 animals had only clusters during add-on treatment, but not during baseline (open symbols). Four of those had reported clusters in their seizure history, two had seizures on sequential days during baseline, and for two, no seizure history was available

Add-on treatment resulted in a mean reduction in MSF, with some animals benefiting with more than 50% reduction in MSF, while others experienced an increase in MSF (Fig. 2). While no animal became seizure free, two animals in cohort A and one in cohort B experienced a reduction by at least 75%. The mean reduction reached 15.46 and 39.41% for cohort A and C, while the reduction reached 35.73%, the difference being significant for cohort B (*p* = 0.043; Table 3). For cohort C, a trend towards reduction (*p* = 0.063) was reached (Table 3). In cohort A, there was one animal, which experienced a series of seizures and cluster seizures following initiation of add-on treatment. After 14 days, the participation of this animal in the study was terminated and the add-on treatment with imepitoin tapered. In this animal, a MSF of 32 was calculated for the short treatment period which is more than 9 times the mean MSF of the whole group during treatment. If this animal was excluded from evaluation, the reduction in MSF was within the same range as seen in the other cohorts, i.e. 27.24% (Table 3, Fig. 3a). MSF reduction was also calculated for the pooled cohort A and B, and for all three cohorts. The MSF reduction for the full analysis set gave a trend towards reduction (*p* = 0.06), while the pooled cohort A + B failed to reach level of significance (*p* = 0.18).

**Table 3:** Decrease in monthly seizure frequency

				95% Confidence Interval			% of animals with	
Variable	Cohort	N	%Decrease	LCL	UCL	*p*-value	any reduction	>50% reduction
MSF	A	16	15.46	−67.03	57.22	0.607	50.0	37.5
	A*	15*	27.24	−38.96	61.90	0.310	53.3	40.0
	**B**	**11**	**35.73**	**1.69**	**57.94**	**0.043**	81.8	36.4
	C	7	39.41	−3.77	64.58	0.063	71.4	42.9
	A + B	27	26.29	−7.36	49.44	0.180	63.0	37.0
	A + B + C	34	27.75	−1.51	48.57	0.060	64.7	38.2
MCD	A	16	−16.77	−161.95	47.95	0.688		
	A*	15*	4.69	−98.58	54.25	0.890		
	B	11	17.88	−46.52	53.98	0.466		
	C	7	8.06	−105.03	58.77	0.806		
	A + B	27	−1.21	−68.03	−64.05	0.962		
	A + B + C	34	0.80	−50.53	34.69	0.969		

MSF = (Number of Seizures/Number of days) x 28 days. MCD = (Number of Cluster days/Number of days) x 28 days. Significant %Decrease (*p* < 0.05) is bolded. In addition, the paired t-test was applied to the corresponding log-transformed data values separately for each cohort and the two combinations of cohorts (i.e., cohort A + B and all 3 cohorts combined). The displayed *p*-value is derived from this paired t-test. The fraction of animals showing any reduction and >50% reduction of MSF (“Responder rate”) is given

*excluding animal No. 6 of cohort A as outlier

An increase in MSF of ≥50% was seen in fife (31.25%) animals of cohort A and in only one animal (9.09%) in cohort B. This increase was not seen in animals in cohort C (Fig. 2, 3b). All five animals of cohort A were receiving potassium bromide (three dogs) or levetiracetam (two dogs) add-on. The animal in cohort B did also receive potassium bromide.

As confirmatory analysis, the data on MSF for cohorts A to C were statistically evaluated via repeated-measures analysis of variance of log-transformed values with main effects for cohort and period and the interaction between cohort and period, followed by Fisher’s least-significant-different comparisons per cohort of the mean difference between the baseline and treatment periods. The analysis revealed that the overall treatment effect for the whole group (cohorts A, B and C combined) was significant (*p* = 0.048), while the difference between the three cohorts was not significant (*p* = 0.068). The treatment effect did not reach level of significance for any of the treatment cohorts individually. The lack of significance for individual cohorts may be related to the low number of animals per cohort.

The add-on treatment had little effect on cluster activity. Some animals had reduced, but others increased MCD (Fig. 3b). In case of cohort A, there was a numerical increase in MCD, but this was again driven primarily by animal no. 6 (Table 3). Five animals of cohort A, two of cohort B, and one of cohort C had no days with cluster activity during the 6 weeks baseline, but during add-on treatment. However, four of these animals had reported cluster seizures in their seizure history, and two had seizures on consecutive days during baseline, which fulfilled the definition for a cluster seizure, but not MCD. For the remaining two animals with new clusters, no seizure history before the baseline period was available. Thus, the initiation of add-on treatment was not associated with occurrence of new clusters.

In addition to the full analysis set, the statistical analysis was repeated for the population of animals which had completed the full treatment period of 24 weeks. There were 5 completers in cohort A, 9 in cohort B, and 5 in cohort C (Table 4). There was no significant difference of baseline MSF and MCD between completer and whole population (MSF completer 3.31 (CI: 2.3-4.75), MSF whole population 3.84, (CI 2.95-4.98); MCD completer 0.72 (CI: 0.48-1.07; MCD whole population 0.65 (0.39-1.10)). Nevertheless, the treatment effect was more pronounced in the completer population, reaching up to 73.4% (*p* = 0.072) reduction in MSF in cohort A, and 51.3% reduction overall (Table 4). The add-on treatment effect reached level of significance for the whole completer population (*p* = 0.002) and the combined completer cohort A + B (*p* = 0.009), while for individual completer cohorts a trend (*p* < 0.1) was observed. In the completer population, a trend towards reduction of cluster activity was observed for pooled cohort A and B (*p* = 0.055), while the cluster activity was increased during the add-on treatment period in cohort C (Table 4).

**Table 4:** Decrease in monthly seizure frequency, completer population

				95% Confidence Interval			% of animals with	
Variable	Cohort	N	%Decrease	LCL	UCL	*p*-value	any reduction	>50% reduction
MSF	A	5	73.42	−20.92	94.16	0.072	80.0	80.0
	B	9	37.12	−2.43	61.36	0.059	88.9	33.3
	C	5	43.56	−14.11	72.06	0.087	80.0	40.0
	**A + B**	**14**	**53.74**	**20.31**	**73.15**	0.009	85.7	50.0
	**A + B + C**	**19**	**51.28**	**26.58**	**67.63**	0.002	84.2	47.4
MCD	A	5	59.75	−78.25	90.92	0.165		
	B	9	25.55	−27.00	56.35	0.239		
	C	5	−20.92	−262.19	59.67	0.656		
	A + B	14	40.25	−1.21	64.73	0.055		
	A + B + C	19	28.04	−12.98	54.21	0.143		

MSF = (Number of Seizures/Number of days) x 28 days. MCD = (Number of Cluster days/Number of days) x 28 days. Significant %Decrease (*p* < 0.05) is bolded. In addition, the paired t-test was applied to the corresponding log-transformed data values separately for each cohort and the two combinations of cohorts (i.e., cohort A + B and all 3 cohorts combined). The displayed *p*-value is derived from this paired t-test. The fraction of animals showing any reduction and >50% reduction of MSF (“Responder rate”) is given.

**Fig. 4:**
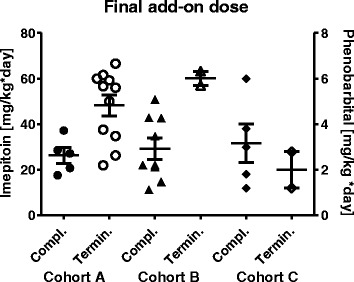
Dose of imepitoin (cohort A and B) and phenobarbital (cohort C) at study termination, separately displayed for animals which completed the full add-on treatment phase of 24 weeks (filled symbols, completer), and animals which terminated early the add-on treatment for various reasons (open symbols, termination). For each sub-cohort, in addition mean ± SEM is given

The study protocol was designed to allow a titration of the add-on medication of imepitoin (cohort A and B), and phenobarbital (cohort C), until a treatment effect was reached, which was considered as adequate. In animals which completed the whole add-on treatment period, the mean daily dose reached was 26.3 ± 3.4 mg/kg per day in cohort A, and 29.3 ± 4.7 mg/kg per day for cohort B. In contrast, the mean dose for the dogs which were terminated early reached 48.3 ± 4.6 and 60.1 ± 3.1 mg/kg per day in cohort A and B, respectively. This increased dose may reflect the insufficient seizure control in the early terminating sub-group, while it at the same time indicates, that for add-on treatment a dose of about 15 mg/kg BID was sufficiently active. In cohort C, the range of doses reached varied widely from 1.2 to 6 mg/kg per day, with no apparent difference between completers and early terminating animals (Fig. 4). At baseline and during add-on treatment, the serum levels of phenobarbital and potassium bromide (where applicable) were determined. In cohort A and B, plasma levels of phenobarbital were mostly within the therapeutic range of 10–35 μg/ml, however in few animals the therapeutic range was exceeded. All animals receiving potassium bromide had plasma levels within the therapeutic range of 0.5 to 2.5 mg/ml. At the end of the add-on treatment period, phenobarbital levels had reached the therapeutic range in cohort C (data not shown). Add on treatment with imepitoin did not significantly change plasma levels of phenobarbital and potassium bromide in cohort A and B.

### Safety

Adverse drug reactions were observed during baseline treatment (retrospective reporting) and during the add-on period. Due to high dose baseline treatment with phenobarbital, in part with add-on potassium bromide or levetiracetam, many ADRs were reported during baseline. In cohort A and B, ataxia and polyphagia were the most frequent ADR during baseline (5 of 27 dogs each), followed by reduced activity (4 of 27). Ataxia was not reported in cohort C during baseline, while polyphagia and reduced activity was reported at a similar rate. Aggressive behavior and restlessness was observed in cohort A and B (3 of 27 dogs), while no aggression, but some restlessness was seen in cohort C. Other ADRs were observed in individual animals only. Following initiation of add-on treatment, the number of ADRs reported increased. Ataxia was the single most frequent ADR, followed by reduced activity. Upon continuation of add-on treatment, the number of ADRs fell again to reach the pre-add-on values. In line with the highest treatment intensity in cohort A, the rate of ADRs was highest in this cohort. There was one additional observation made: in some patients treated add-on with imepitoin, owners reported that animals became more lively as compared to the baseline period. While this is likely a drug-induced finding, it was not categorized as ADR.

Five animals were euthanized on owner’s request during the course of the study, i.e. prior to conclusion of the 24 weeks add-on treatment period. All 5 patients were from cohort A. The primary reason for euthanasia was lack of efficacy/reduced quality of life. While it is notable that all euthanized animals were from cohort A, this correlated with the observed increased baseline severity of epilepsy and increased treatment intensity in this cohort.

The add-on treatment was without effect on haematological parameters. At baseline, the majority of animals undergoing phenobarbital treatment in cohorts A and B had liver enzyme levels within the normal range, but a fraction of animals had in part strongly elevated liver enzymes, alkaline phosphatase being the most sensitive parameter. Upon initiation of add-on treatment with imepitoin, there was no change in liver enzyme levels. In animals undergoing imepitoin treatment at baseline, all animals were within the normal range for all liver enzymes. Upon initiation of low dose phenobarbital treatment, there was no change in liver enzyme levels. For both urea and creatinine, there were no clinical relevant deviations from normal range throughout the study.

## Discussion

In approximately 20-40% of the dogs with idiopathic epilepsy under treatment, seizure reduction may not be adequate using only one AED [3]. One strategy to overcome drug resistance is to combine two or more antiepileptic treatments. Imepitoin is currently not approved for combination treatment with other AEDs. So far, only one study [12, 15] evaluated the safety and efficacy of imepitoin administered add-on to phenobarbital, and compared the results with add-on treatment with potassium bromide. In this study, only patients with monotherapy of phenobarbital were included. The effect of imepitoin add-on to phenobarbital was similar to the effect of potassium bromide, but the study population was small [12, 15].

The aim of the current study was to get further insight into the safety and efficacy of a combination of phenobarbital with imepitoin. To best reflect the clinical situation, patients receiving more than one baseline medication were allowed to be included in this study. Thus, patients with phenobarbital with or without add-on potassium bromide or levetiracetam were included. The diagnosis of idiopathic epilepsy was verified using not only clinical criteria and blood work, but also MRI and of CSF analysis, to exclude structural epilepsy or reactive seizures, in accordance with the current recommendations (tier II confidence level) [2]. The study was designed as open-label monocentric cohort study. No separate control group was included. While the lack of a comparator control group prevented the statistical evaluation of placebo effects and fluctuations of seizure activity over time, an attempt to compensate for this deficit was made through use of a defined baseline period of 6 weeks, with diligent seizure recordings using a seizure diary, to form the basis for an individual patient pre-post comparison.

The three cohorts included in the study were not different with regard to gender, breed, or body weight. Several breeds and mixed breeds were included. Australian Shepherds and Border Collies, both known for epilepsy predisposition with severe seizures [4, 5], were most frequently represented, contributing together about one third of the patients. However, the three cohorts tended to differ in their baseline characteristics. Animals included in cohort A had a tendency to suffer from more severe epilepsy as compared to cohort B and C, as reflected by a longer duration of epilepsy, highest MSF, rate of animals with cluster seizure activity and status epilepticus, dose of phenobarbital, rate of animals with potassium bromide or permanent levetiracetam in baseline, and rate of reported ADRs during baseline. The individual differences however did not reach level of significance. Animals in cohort C had the shortest duration of epilepsy, but had a high rate of cluster activity during baseline. The reason for this difference is not entirely clear. One possible explanation is that initially, when cohort A was started, cases of drug-resistant patients which had unsuccessfully explored add-on treatment with potassium bromide or levetiracetam were included, while upon start of cohort B and C, most patients included had failed only their first antiepileptic therapy. For those, the treating veterinarian could choose to participate in the study or try potassium bromide or levetiracetam. Indeed, in cohort A 56.3% of animals had a 2^nd^ permanent antiepileptic medication during baseline, while only 27.3% of patients in cohort B had this 2^nd^ AED in baseline. These differences in baseline medication and underlying disease characteristics need to be taken into consideration if cohorts are compared. Bromides are known for their narrow margin of safety [16]. The most common adverse effects seen with potassium bromide with or without phenobarbital combination treatment are polydipsia, polyphagia, and increased lethargy [16]. The high frequency of dogs treated with potassium bromide in cohort A may be the reason why the rate of adverse drug reactions is highest in cohort A during baseline. The rate of dogs with cluster seizures during baseline was higher in cohort A compared to cohort B. The tendency to develop cluster seizures or status epilepticus has been found to negatively influence the treatment outcome in dogs with epilepsy [17, 18]. Thus, animals in cohort A can be expected to be more treatment resistant as compared to cohort B.

Treatment of imepitoin add-on to phenobarbital resulted in a reduction of the MSF by about one third. The reduction of MSF was enlarged, if only the completer population of the pooled cohorts A and B was evaluated. This effect is identical in frequency and effect size to the effect of potassium bromide added to phenobarbital in a previous study [19]. The authors report for those animals followed for 1 year after initiation of potassium bromide therapy a reduction in seizure frequency in 83% of dogs, and on average, a 53% reduction in the number of seizures compared with the previous 12 months.

The add-on treatment had no clinically relevant effect on cluster activity. Neither the frequency of cluster seizures, nor the fraction of animals suffering from cluster seizures was changed following initiation of add-on treatment with imepitoin or phenobarbital. The initiation of add-on treatment was not associated with occurrence of new clusters. Neither treatment of imepitoin add-on to phenobarbital with or without potassium bromide or levetiracetam, nor treatment of phenobarbital add-on to imepitoin with or without levetiracetam had a significant influence on MCD. In fact, cluster seizures and younger age at onset haven been identified as predictors for treatment resistance [5, 17, 18, 20]. However, individual animals benefited from the add-on treatment, with more than 50% reduction in cluster activity (Fig. 3B).

Aggravation of seizure activity was primarily seen in cohort A, with only one case in cohort B. The underlying cause for this aggravation is not known. All five animals experiencing ≥50% increase in MSF were receiving potassium bromide (three dogs) or levetiracetam (two dog) add-on. In patients receiving potassium bromide, three drugs (phenobarbital, imepitoin, KBr) are administered which all act at the GABAergic synapse. While it is well understood, that benzodiazepines and barbiturates are acting synergistically on the GABAergic synapse [21], little is known about how potassium bromide, in part replacing the chloride influx, interacts as a 3^rd^ modulator. Since three other animals with potassium bromide add-on in baseline and one animal with levetiracetam add-on in baseline were experiencing a ≥50% reduction in MSF, one can conclude, that potassium bromide is not per se a predictor of seizure aggravation. Changes in seizure frequency are a common feature of the disease. Even in the study evaluating potassium bromide add-on to phenobarbital, only 83% of dogs had nominally reduced MSF, leaving 17% of animals with seizure aggravation [19]. In cohort B, using a low starting dose of imepitoin, only one out of 11 dogs showed a >50% increase in MSF. This indicates that combination of phenobarbital and imepitoin is well possible. Seizure aggravation was limited to only a few cases when using a low starting dose of imepitoin, but this finding should be explored in a larger cohort.

The pharmacological effect of imepitoin was also evaluated separately in the completer population. These patients represent a population where the owners were willing to continue the add-on treatment, indicating that the combination may have had a positive effect on quality of life of their dogs, albeit this was not evaluated. In this population, a dose of about 15 mg/kg BID imepitoin was administered, resulting in a significant mean reduction of 53.7%, with 85.7% of dogs showing a nominal reduction in MSF. The dose escalation was not limited by safety issues, since in animals with insufficient seizure control the dose was safely raised to up to 30 mg/kg BID. The titration results underscore that imepitoin is a potent anticonvulsant in responder animals, while other animals remain treatment resistant even with the highest dose applied.

The phenobarbital add-on dose was found to be in the lower therapeutic range of 3.2 ± 0.84 mg/kg per day in cohort C (about 1.6 mg/kg BID), which is less than half the dose of phenobarbital administered in cohort A and B as baseline medication. This low dose of phenobarbital was sufficient to reduce the MSF in 5 out of 7 evaluated dogs. The use of phenobarbital add-on to imepitoin may thus allow a lower dose of phenobarbital and may result in a long-term safety advantage, compared to high-dose phenobarbital monotherapy. However, the results in cohort C indicate, that low dose phenobarbital treatment add-on to imepitoin was not capable of reducing the cluster seizure activity in imepitoin-resistant dogs.

## Conclusion

The data indicate that combination treatment of imepitoin and phenobarbital is well tolerated in dogs with drug-resistant epilepsy. The efficacy results must be interpreted cautiously in view of the lack of adequate placebo control and randomization. While not all animals benefit from the combination treatment, a clinically meaningful reduction in seizure frequency of ≥50% was obtained in 36-42% of all animals. The effect size in animals completing the study was comparable to the effect size of potassium bromide reported by others, but potassium bromide is less well tolerated than imepitoin. The lower starting dose of 5 mg/kg BID imepitoin was found to be better tolerated than the higher starting dose of 10 mg/kg BID, and an up-titration to on average 15 mg/kg BID was sufficient. Neither imepitoin nor phenobarbital add-on treatment was capable of suppressing cluster seizure activity, making cluster seizure activity an important predictor for drug-resistance.

## Abbreviations

ADR: Adverse drug reaction (with possible or likely treatment relation)
AED: Antiepileptic drug
BID: Twice daily
CI: Confidence interval
CSF: Cerebrospinal fluid
GABA: γ-Amino butyric acid
GM: Geometric mean
MRI: Magnetic resonance imaging
MSC: Monthly frequency of days with cluster seizures
MSF: Monthly seizure frequency
